# Diseases of the Nervous System

**Published:** 1906-03-10

**Authors:** 


### PROGRESS IN MEDICINE AND SURGERY.

### DISEASES OF THE NERVOUS SYSTEM.

(*Continued from page* 353.)

**Cerebral Arteriosclerosis.**—Notwithstanding the facts that arteriosclerosis of the cerebral vessels is not uncommon in various forms of mental disease, especially in later life, and that on the other hand it may undoubtedly be present without causing any recognisable disease of the mind, A. M. Barrett[5] thinks there is sufficient ground for establishing as a distinct entity an arteriosclerotic dementia. This group shows clinically a prominence of symptoms referable to focal or diffuse vascular brain disease which are found anatomically to be associated with destructive lesions of the brain due to diseased vessels. In those familiar cases of dementia due to such coarse lesions as hæmorrhage and softening, and described as post-paralytic, post-apoplectic, and arteriosclerotic, it will usually be found that an arteriosclerotic condition of the finer as well as of the larger vessels is present, together with more or less destruction of the cortical nervous elements. Alzheimer in 1902 described four groups of cases in which the mental process is associated with arteriosclerotic brain disease, all, however, as Barrett points out, being varieties of one process. Group I. consists in arteriosclerotic brain atrophy, with symptoms either mild or severe. In the milder cases pronounced increase of mental fatigue, memory weakness, headache, and dizzy attacks are observed, and on examination severe sclerosis of the arteries is revealed with some overgrowth of neuroglial tissue, but no gross changes. In the more severe form the symptoms are graver and progress to deep dementia, and epileptic convulsions or apoplectic attacks occur. Group II. consists of subcortical encephalitis, an atrophy of the deep-lying white substance due to sclerosis of the long medullary arteries. It has been specially described by Binzwanger. Interference with the association processes is the first and most striking symptom. Speech is affected early, and there are various aphasic disturbances with mental confusion and convulsive attacks. In Group III. there is a perivascular gliosis and consequent atrophy of nervous elements in areas of which the nutrition is impaired by reason of the sclerosis of the arteries supplying the parts. In Group IV. senile brain atrophy is present, showing as a disintegration of the nervous elements in peculiar wedge-shaped foci, or in streaks, and due to an arteriosclerotic degeneration of the smaller vessels of the cortex. In the production of these types, which are usually somewhat obscured by the occurrence of focal lesions, two processes must be considered—the changes in the vessels and the reactions in the nervous tissue. In the larger vessels of the brain the changes are similar to those found in arteriosclerotic vessels elsewhere. The intima is chiefly affected, becoming irregularly thickened by proliferation of cells and elastic tissue by fibrous new formation. Later, necrotic degenerative processes lead to atheromatous degeneration, or more rarely hyaline transformation. In the finer vessels the endothelial cells proliferate and the elastic fibres multiply or split, leading sometimes to occlusion of the original lumen of the vessel and the formation in it of new channels; as many as four new channels have been observed running through the proliferated endothelium. The cross section of such vessels gives the appearance of clusters or chains of rings. In the nerve tissue, as a result of these changes, focal softenings occur from the sudden shutting off of nutrition, or in cases of more gradual deprivation partial softening and degeneration of the less resistant nervous elements with overgrowth of the glia. The hyaline degeneration leads to fine miliary aneurysms, which by rupture produce the larger cerebral hæmorrhages. Epithelioid cells are usually present in arteriosclerotic foci. Arteriosclerosis may be present as a purely associated condition in general paralysis, and in senile atrophy. From the former it is to be histologically differentiated by the less diffuse character of the process, and above all by the absence of lymphoid and plasma cell infiltration of the vessel wall. In senile atrophy, which may occur without arteriosclerosis, the disappearance of the nerve cells is also more diffuse and general, while in the dementia due to vascular change the cortex shows a patch-like degeneration, and the lesions are focal in character and related to the distribution of the arteries. In senile arteriosclerotic dementia there are observable, clinically, among other symptoms, forgetfulness, loss of judgment, delusions of persecution, and apathy deepening into a profound dementia. These symptoms are common in non-vascular senile dementia, but in addition, where the vessels are at fault, are observed "shocks," focal paralysis, speech defects, articulatory and aphasic, and the constitutional signs of arteriosclerosis. Characteristics of the disease as described in Group I. are weakness of the retentive memory—an early symptom—and marked dullness and lack of productivity, rapid alternation of moods, but no constant depression; all moods tending decidedly to marked apathy and indifference to surroundings. In many respects these cases very closely resemble general paralysis, with which, indeed, they may be associated. Special points to be noted are the absence of delusions, especially of the expansive type, and the striking unproductiveness of the mental attitude, the general aspect being one of apathy or emptiness. C. K. Mills and C. D. Camp [6] have recorded the case of a woman, aged 63, in whom advanced arteriosclerosis was found in different parts of the brain. The patient had suffered from impaired vision for two years, when a sudden cerebral attack was followed by complete blindness of the right eye and greatly restricted vision in the left, together with some mental excitement and loss of power of co-ordination, so that she could not stand. Visual hallucinations were prominent, and mania developed before death, which occurred in about three months. The autopsy revealed chronic interstitial nephritis, chronic and acute endocarditis, cardiac hypertrophy, fatty liver, and a high degree of atheroma of the vessels at the base of the brain. No areas of softening were found, but the microscope revealed a general thickening of the cortical vessels and enlargement of the perivascular spaces, but no round-celled infiltration of these spaces nor of the pia. In the lateral parts of the occipital lobes, and on either side of the calcarine fissure, were areas of intense congestion, showing the formation of numerous very fine new capillaries. The pyramidal nerve-cells in these areas and the white fibres beneath showed some degeneration. A sister of this patient, a lady aged 64, died previously after many years of mental peculiarity, ending finally in dementia. Amnesia was a prominent symptom, not only for names, but also for recent events. Here also the vessels of the base were atheromatous, and the pial vessels showed numerous miliary aneurysms, while areas of softening, myelin degeneration, and cellular changes were also found.

[5] American Jour. of Insanity, July. [6] Ibid.

